# Correction: Ben Ammar et al. Anti-Inflammatory Activity of Geraniol Isolated from Lemon Grass on Ox-LDL-Stimulated Endothelial Cells by Upregulation of Heme Oxygenase-1 via PI3K/Akt and Nrf-2 Signaling Pathways. *Nutrients* 2022, *14*, 4817

**DOI:** 10.3390/nu16050596

**Published:** 2024-02-22

**Authors:** Rebai Ben Ammar, Maged Elsayed Mohamed, Manal Alfwuaires, Sarah Abdulaziz Alamer, Mohammad Bani Ismail, Vishnu Priya Veeraraghavan, Ashok Kumar Sekar, Riadh Ksouri, Peramaiyan Rajendran

**Affiliations:** 1 Department of Biological Sciences, College of Science, King Faisal University, Al-Ahsa 31982, Saudi Arabia; malfwuaires@kfu.edu.sa (M.A.); salamer@kfu.edu.sa (S.A.A.); 2 Laboratory of Aromatic and Medicinal Plants, Center of Biotechnology of Borj-Cedria, Technopole of Borj-Cedria, P.O. Box 901, Hammam-Lif 2050, Tunisia; ksouri.riadh@gmail.com; 3 Department of Pharmaceutical Sciences, College of Clinical Pharmacy, King Faisal University, Al-Ahsa 31982, Saudi Arabia; memohamed@kfu.edu.sa; 4 Department of Pharmacognosy, Faculty of Pharmacy, University of Zagazig, Zagazig 44519, Egypt; 5 Department of Basic Medical Sciences, School of Medicine, Aqaba Medical Sciences University, Aqaba 11191, Jordan; m.bani0331@gmail.com; 6 Centre of Molecular Medicine and Diagnostics (COMManD), Department of Biochemistry, Saveetha Dental College & Hospitals, Saveetha Institute of Medical and Technical Sciences, Saveetha University, Chennai 600077, Tamil Nadu, India; vishnupriya@saveetha.com; 7 Centre for Biotechnology, Anna University, Chennai 600025, Tamil Nadu, India; ashokkumar@annauniv.edu

## Error in Figure

In the original publication [1], there was a mistake in Figure 5 as published by mistake in image position. The corrected Figure 5 appears below.

**Figure 5:**
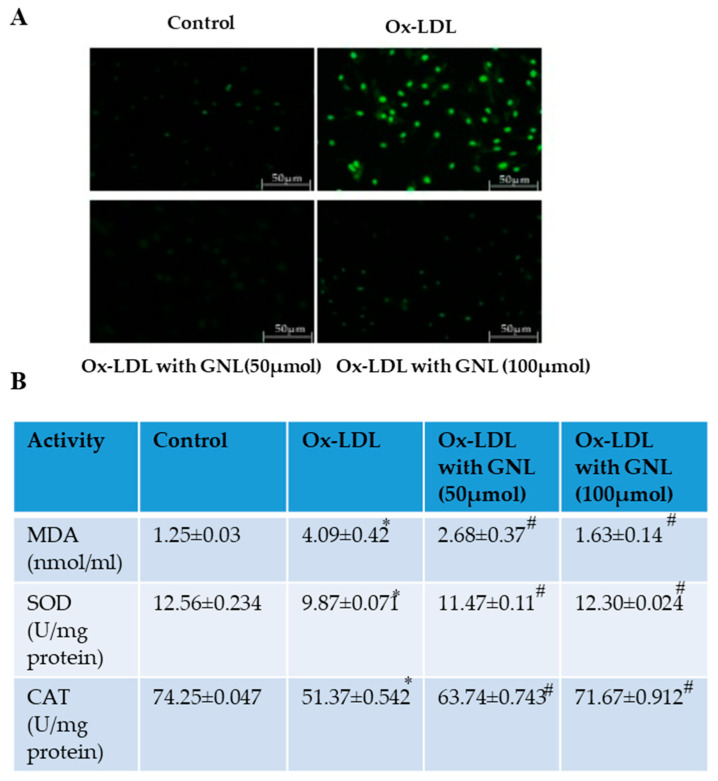
Effect of GNL on Ox-LDL-induced ROS production in HUVECs. (**A**) The HUVECs were then pretreated with GNL (0, 50 and 100 µM, for 2 h), followed by Ox-LDL (100 µg/mL) for 24 h. We measured the intracellular ROS levels using DCF fluorescence. (**B**) LPO, SOD and CAT. Based on the manufacturer’s instructions, we used ELISA kits. There are three replicates of each value, and * represents *p* < 0.05; thus, there is a significant difference when compared to the control group. The # represents *p* < 0.05; thus, there are significant differences between the Ox-LDL alone and GNL with Ox-LDL treatment groups.

In the original publication, there was a mistake in Figure 6 as published by mistake in the Beta-actin image. The corrected Figure 6 appears below.

**Figure 6:**
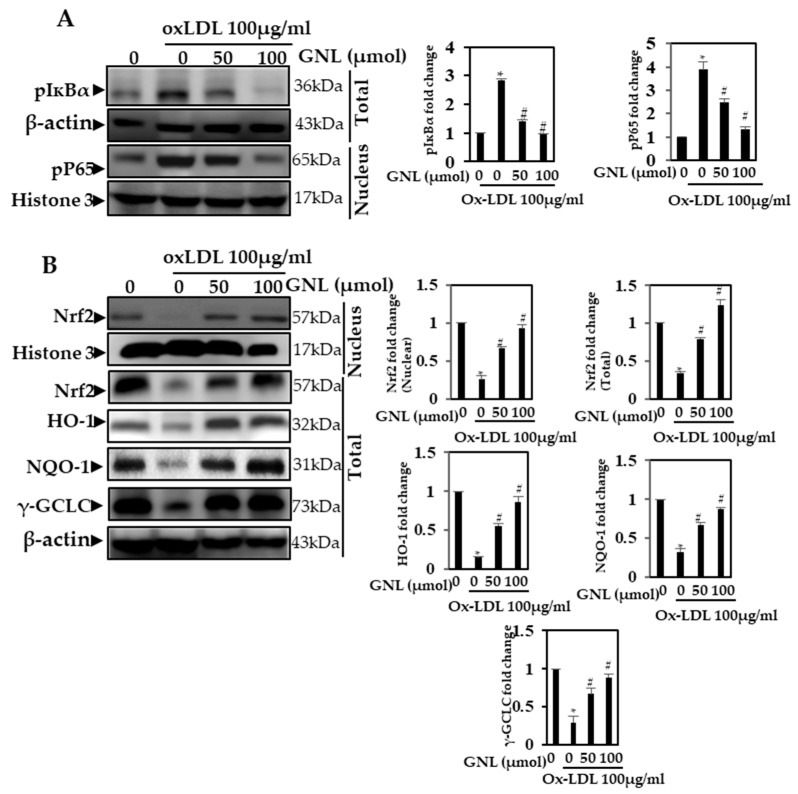
NF-ᴋB p65 expression is affected by GNL. (**A**) p-IᴋBα and pNF-ᴋB p65 antibodies were used to detect nuclear protein extract and total protein extract on 10–12% SDS-PAGE (polyacrylamide gel electrophoresis). (**B**) By Western blot analysis, we measured the levels of nuclear Nrf2 and NQO-1, HO-1 and γ-GCLC. There are three replicates of each value, and * represents *p* < 0.05; thus, there is a significant difference when compared to the control group. The # represents *p* < 0.05; thus, there are significant differences between the Ox-LDL alone and GNL with Ox-LDL treatment groups.

The authors apologize for any inconvenience caused and state that the scientific conclusions are unaffected. This correction was approved by the Academic Editor. The original publication has also been updated.
