# Supplementary material for: Anti-Inflammatory Activity of Geraniol Isolated from Lemon Grass on Ox-LDL-Stimulated Endothelial Cells by Upregulation of Heme Oxygenase-1 via PI3K/Akt and Nrf-2 Signaling Pathways
Source: Nutrients. 2022 Nov 14;14(22):4817. doi: 10.3390/nu14224817 (PMC9695721; doi:10.3390/nu14224817)
Supplement: Supplementary file 1 [file nutrients-14-04817-s001.zip › nutrients-1994945-supplementary.pdf]

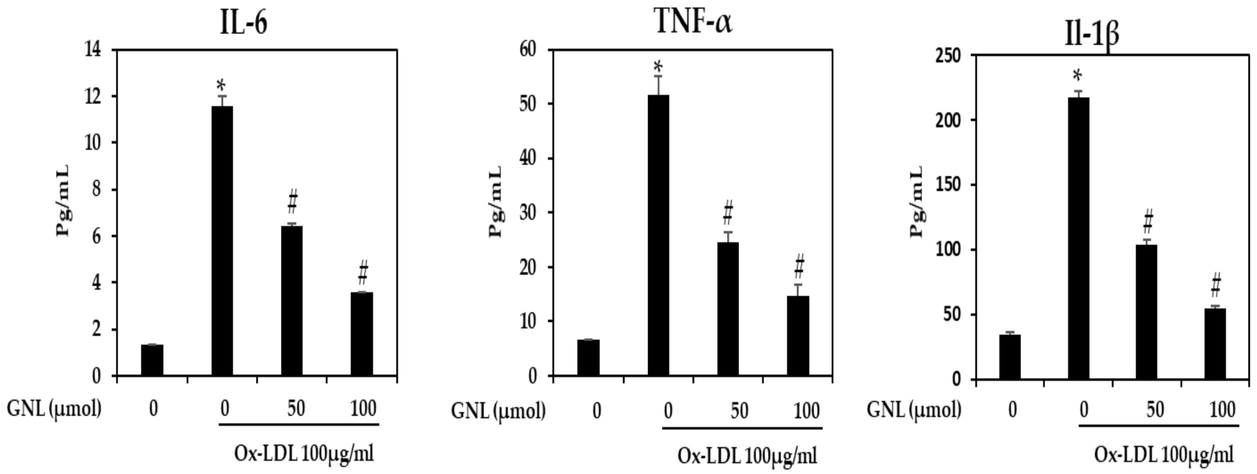

Figure S1. GNL mitigated Ox-LDL-induced cytokine production in endothelial cells. HUVECs cells were treated with the GNL and/or Ox-LDL for 24 h. Level of TNF-α,IL-6 and IL-1β, was assessed by ELISA. There are three replicates of each value, and * $p<0.05$ represents a significant difference when compared to a control group. The #$p<0.05$ represents significant differences between Ox-LDL alone and GNL with Ox-LDL treatment groups.

SUPPLEMENTARY TABLES

Table S1. Antibody details of the proteins used in immunoblot analysis

Antibody	Molecular weight (kDa)	Company catalogue	Dilution
**TNF-α**	17	(#PA5-19810) Thermo Fisher Scientific, Inc. (Waltham, MA, USA)	1:5000
**IL-6**	21	#P620 Thermo Fisher Scientific, Inc. (Waltham, MA, USA)	1:5000
**IL-1β**	17	(#PBOIL1BI) Thermo Fisher Scientific, Inc. (Waltham, MA, USA)	1:5000
**ICAM-1**	72	(#MA5407) Thermo Fisher Scientific, Inc. (Waltham, MA, USA)	1:5000
**VCAM-1**	98	# PA5-87406 Thermo Fisher Scientific, Inc. (Waltham, MA, USA)	1:3000
**HO-1**	32	(#PA5-77833) Thermo Fisher Scientific, Inc. (Waltham, MA, USA)	1:5000
**NQO-1**	31	(#PA5-82294 Thermo Fisher Scientific, Inc. (Waltham, MA, USA)	1:1000
**γ-GCLC**	73	(#PA5-44190), Thermo Fisher Scientific, Inc. (Waltham, MA, USA)	1:5000
**p-P65**	65	(#MA5-15160), Thermo Fisher Scientific, Inc. (Waltham, MA, USA)	1:2000
**p-IKBα**	36	(#PA5-36653) Thermo Fisher Scientific, Inc. (Waltham, MA, USA)	1:2000
**FABP4**	15	PA5-30591 Thermo Fisher Scientific, Inc. (Waltham, MA, USA)	1:2000
**TGF-β-1**	13	# **MA5-23795** Thermo Fisher Scientific, Inc. (Waltham, MA, USA)	1:1000
**pPI3K**	85	PA5-104853 Thermo Fisher Scientific, Inc. (Waltham, MA, USA)	1.2000
**PI3K**	85	#PA5-29220 Thermo Fisher Scientific, Inc. (Waltham, MA, USA)	1:2000
**pAKT**	60	Cat #44-602G Thermo Fisher Scientific, Inc. (Waltham, MA, USA)	1:5000
**AKt**	60	Cat #44-609G Thermo Fisher Scientific, Inc. (Waltham, MA, USA)	1:2000

**Nrf2**	62	(AB-M-018) MOLEQULE-ON (New Lynn, Auckland, New Zealand)	1:5000
**secondary antibodies goat anti-rabbit**	-	(AB-M-010) MOLEQULE-ON (New Lynn, Auckland, New Zealand)	1:10,000
**secondary goat anti-mouse**	-	(AB-M-009) MOLEQULE-ON (New Lynn, Auckland, New Zealand)	1:10,000
**β-actin**	43	(AB-M-003) MOLEQULE-ON (New Lynn, Auckland, New Zealand)	1:10,000

Table S2. Primer sequences

Gene	Sequences
IL-6	Forward: GGTACATCCTCGACGGCATCT<br>Reverse: GTGCCTCTTTGCTGCTTTCAC
VCAM-1	Forward: CAAATCCTTGATACTGCTCATC<br>Reverse: TTGACTTCTTGCTCACAGC
ICAM-1	Forward: TATGGCAACGACTCCTTCT<br>Reverse: CATTCAGCGTCACCT TGG
β-Actin	Forward: CTGGCACCACACCTTCTACAATGAGC<br>Reverse: GAGGATCTTCATGAGGTAGTCAGTC
